# Lower limb joint motion and muscle force in treadmill and over-ground exercise

**DOI:** 10.1186/s12938-019-0708-4

**Published:** 2019-08-22

**Authors:** Jie Yao, Ning Guo, Yanqiu Xiao, Zhili Li, Yinghui Li, Fang Pu, Yubo Fan

**Affiliations:** 1 Key Laboratory for Biomechanics and Mechanobiology of Ministry of Education, School of Biological Science and Medical Engineering, Beihang University, Beijing, 100083 People’s Republic of China; 2 Beijing Advanced Innovation Centre for Biomedical Engineering, Beihang University, Beijing, 100083 People’s Republic of China; 3 Department of Orthopaedics and Traumatology, Li Ka Shing Faculty of Medicine, University of Hong Kong, Hong Kong, 999077 People’s Republic of China; 4 State Key Laboratory of Space Medicine Fundamentals and Application, China Astronaut Research and Training Center, Beijing, 100094 People’s Republic of China; 5 National Research Center for Rehabilitation Technical Aids, Beijing, 100176 People’s Republic of China

**Keywords:** Treadmill, Over-ground, Motion capture, Muscle force, Stride frequency

## Abstract

**Background:**

Treadmill exercise is commonly used as an alternative to over-ground walking or running. Increasing evidence indicated the kinetics of treadmill exercise is different from that of over-ground. Biomechanics of treadmill or over-ground exercises have been investigated in terms of energy consumption, ground reaction force, and surface EMG signals. These indexes cannot accurately characterize the musculoskeletal loading, which directly contributes to tissue injuries. This study aimed to quantify the differences of lower limb joint angles and muscle forces in treadmills and over-ground exercises. 10 healthy volunteers were required to walk at 100 and 120 steps/min and run at 140 and 160 steps/min on treadmill and ground. The joint flexion angles were obtained from the motion capture experiments and were used to calculate the muscle forces with an inverse dynamic method.

**Results:**

Hip, knee, and ankle joint motions of treadmill and over-ground conditions were similar in walking, yet different in running. Compared with over-ground running, joint motion ranges in treadmill running were smaller. They were also less affected by stride frequency. Maximum Gastrocnemius force was greater in treadmill walking, yet maximum Rectus femoris and Vastus forces were smaller. Maximum Gastrocnemius and Soleus forces were greater in treadmill running.

**Conclusions:**

Treadmill exercise results in smoother joint kinematics. In terms of muscle force, treadmill exercise requires lower loading on knee extensor, yet higher loading on plantar flexor, especially on Gastrocnemius. The findings and the methodology can provide the basis for rehabilitation therapy customization and sophistic treadmill design.

**Electronic supplementary material:**

The online version of this article (10.1186/s12938-019-0708-4) contains supplementary material, which is available to authorized users.

## Background

Walking and running are commonly used physical activities and rehabilitation therapies; they can effectively promote the neuromotor and cardiorespiratory functions [1–4]. Because of the convenience and controllability, treadmill exercise has become a common alternative to over-ground walking or running. It has been widely used in daily fitness, clinical rehabilitation, sport biomechanical research, and even astronaut training in space [5–7]. However, growing researches have reported the differences between treadmill and over-ground exercises. For example, at the same speed, the treadmill running has smaller step length and knee flexion angle than over-ground running [8, 9], yet it requires more metabolic energy [10, 11]. Furthermore, treadmill could provide a stable and uniform condition, thus causes less impulse and center of mass excursion. However, the vertical ground reaction force (GRF) in treadmill and over-ground running are similar [12]. The causes of the above phenomena are multifactorial and include the neuromodulation strategy and external loading conditions [13–16]. However, the quantitative mechanism remains unclear.

The differences in the two walking or running patterns may influence the effect of the exercise and could result in negative or positive impact on the physiological system of people, especially on patients. Previous study reported that treadmill training can lower the energy cost of cerebral palsy gait, thus may enhance functional mobility in cerebral palsy patients [17]. Recent study also suggested that the treadmill walking provided a more regularized gait than the over-ground one, and could be incorporated into a therapeutic protocol for patients with Rett syndrome [18]. However, in patients with stroke and lower limb amputations, the energy cost of treadmill exercise is significantly greater than that of over-ground exercise [19, 20], which implied that over-ground exercise is more beneficial for the rehabilitation of such patients. Understanding the impact of walking and running conditions on the spatiotemporal loading distributions in musculoskeletal system can provide basis for the assessment of exercise as a therapeutic modality and the customization of personalized exercise strategy.

Many scientific researches regarding foot–ground interaction and surface electromyography (EMG) have been carried out on treadmill and over-ground exercise. It was reported that the vertical GRF of treadmill and over-ground running were similar, while the anterior–posterior GRF were different [21, 22]. EMG signals in treadmill and over-ground running modes were also collected to reflect the modular control of muscle activation [23, 24]. These indexes can provide clues for understanding the human body kinetics, but they cannot characterize the spatiotemporal loading on musculoskeletal system, which directly contributes to the tissue injuries.

This study aimed to compare the differences of lower limb joint angles and muscle forces in treadmills and over-ground exercises. The stride frequency was used as the control condition. Walking at 100 and 120 steps/min and running at 140 and 160 steps/min were analyzed. Joint motion was measured with the inertial-based motion capture methods and was used to estimate the muscle forces with the inverse dynamic algorithm method.

## Results

### Joint flexion angles in gait cycle

**Fig. 1:**
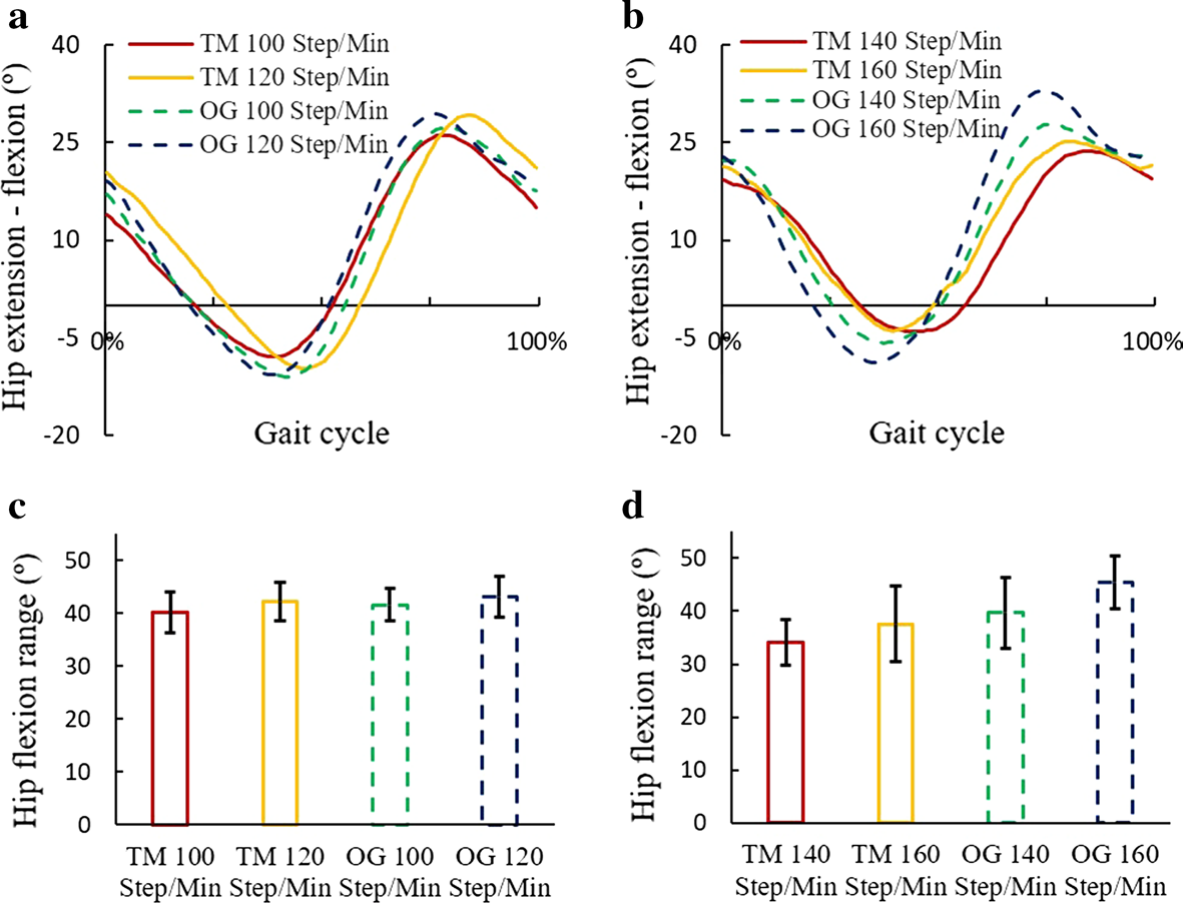
Hip flexion angles in treadmill and over-ground exercises. **a** Hip flexion angles during walking at 100 and 120 steps/min. **b** Hip flexion angles during running at 140 and 160 steps/min. **c** Sagittal motion range of hip joint in walking. **d** Sagittal motion range of hip joint in running

**Fig. 2:**
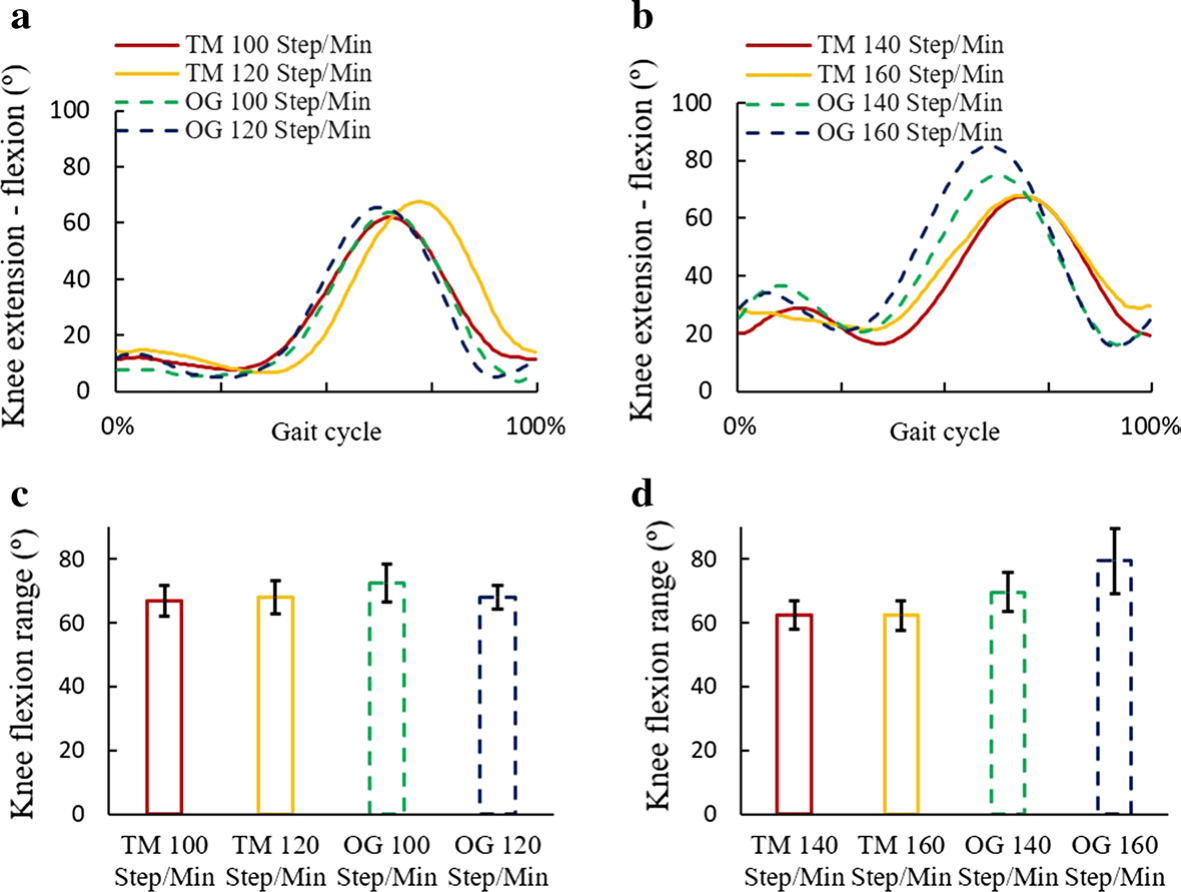
Knee flexion angles in treadmill and over-ground exercises. **a** Knee flexion angles during walking at 100 and 120 steps/min. **b** Knee flexion angles during running at 140 and 160 steps/min. **c** Sagittal motion range of knee joint in walking. **d** Sagittal motion range of knee joint in running

**Fig. 3:**
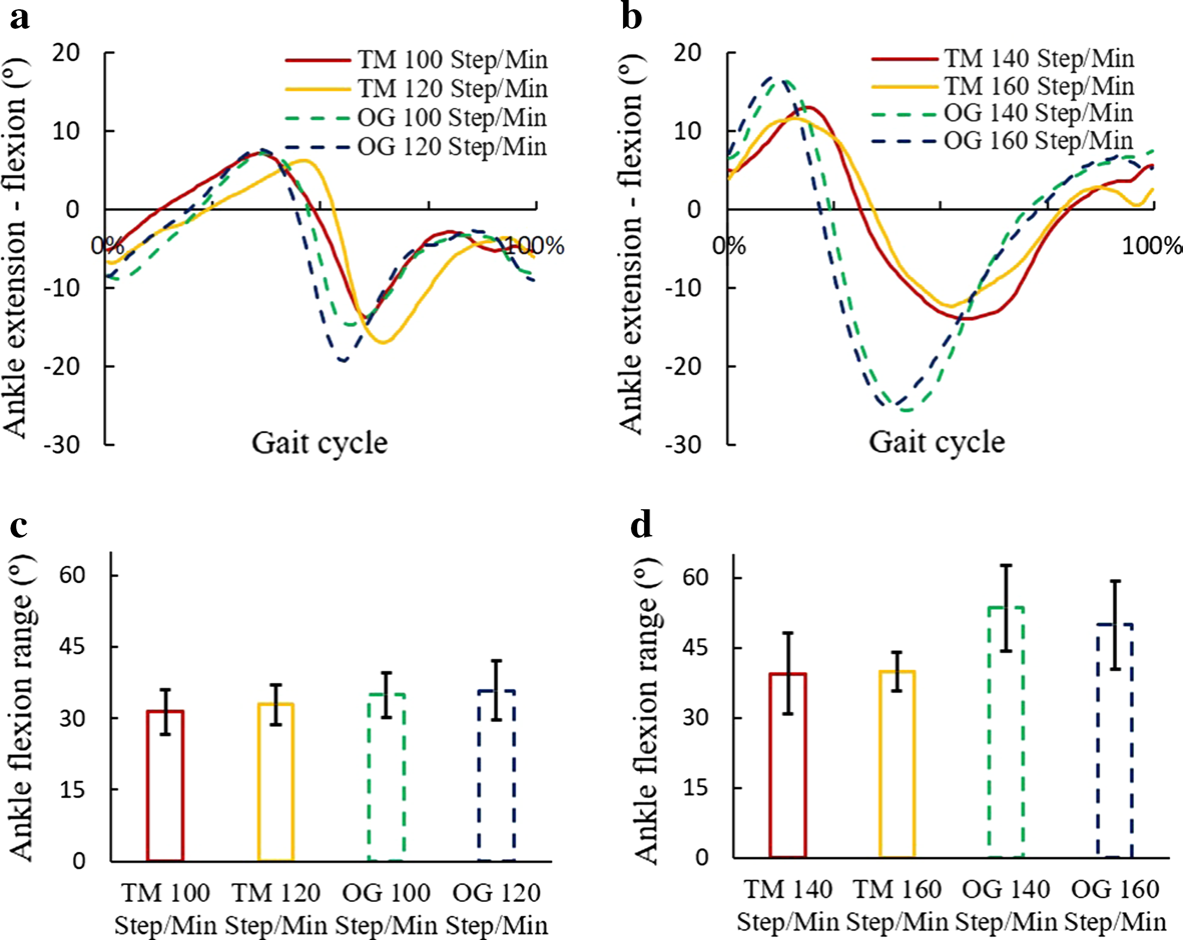
Ankle flexion angles in treadmill and over-ground exercises. **a** Ankle flexion angles during walking at 100 and 120 steps/min. **b** Ankle flexion angles during running at 140 and 160 steps/min. **c** Sagittal motion range of ankle joint in walking. **d** Sagittal motion range of ankle joint in running

Joint flexion angles of treadmill and over-ground conditions were similar in walking, yet different in running (Figs. 1, 2, 3). Compared with the over-ground running, the ranges of hip, knee, and ankle motion in treadmill running were smaller, and the peak flexion angles occurred later in the gait cycle. In the treadmill running, hip motion range increased with the stride frequency, yet knee and ankle motion ranges changed slightly. In over-ground running, hip and knee motion ranges increased with the stride frequency, yet ankle motion range decreased. In the walk-to-run transition, the motion range of hip joint decreased, while the motion range of ankle joint increased, particularly in over-ground running. The averages and standard deviations of the joint motions in treadmill and over-ground motions at each stride frequency are shown in Additional file 1.

### Correlation between muscle force and EMG

The timings of the peak muscle forces and the peak EMG signals in the gait cycle are shown in Table 1. A significant correlation was found between them (Spearman correlation coefficient was 0.514*, *P *= 0.03), which provided a validation of the muscle force calculation. Furthermore, the *ICC (2,k)* results of the muscle forces were greater than 0.75 (the least *ICC(2,k)* was 0.94 for Rectus force in the over-ground running at 140 steps/min), which indicate a desirable repeatability of the methodology.

**Table 1:** The timings of peak muscle forces and peak EMG signals in the gait cycle [force timing % (EMG timing %)]

	Stride frequency	Gastrocnemius	Rectus	Soleus	Vastus
Treadmill	100	38% (22%)	56% (13%)	49% (23%)	56% (55%)
	120	37% (31%)	51% (27%)	47% (32%)	57% (63%)
	140	27% (27%)	43% (10%)	22% (28%)	5% (9%)
	160	23% (86%)	39% (18%)	21% (36%)	6% (19%)
Over-ground	100	34% (38%)	54% (38%)	44% (38%)	54% (42%)
	120	31% (17%)	46% (39%)	38% (17%)	48% (44%)
	140	17% (20%)	49% (19%)	17% (19%)	4% (12%)
	160	20% (16%)	34% (20%)	20% (16%)	5% (9%)

### Muscle forces in gait cycle

**Fig. 4:**
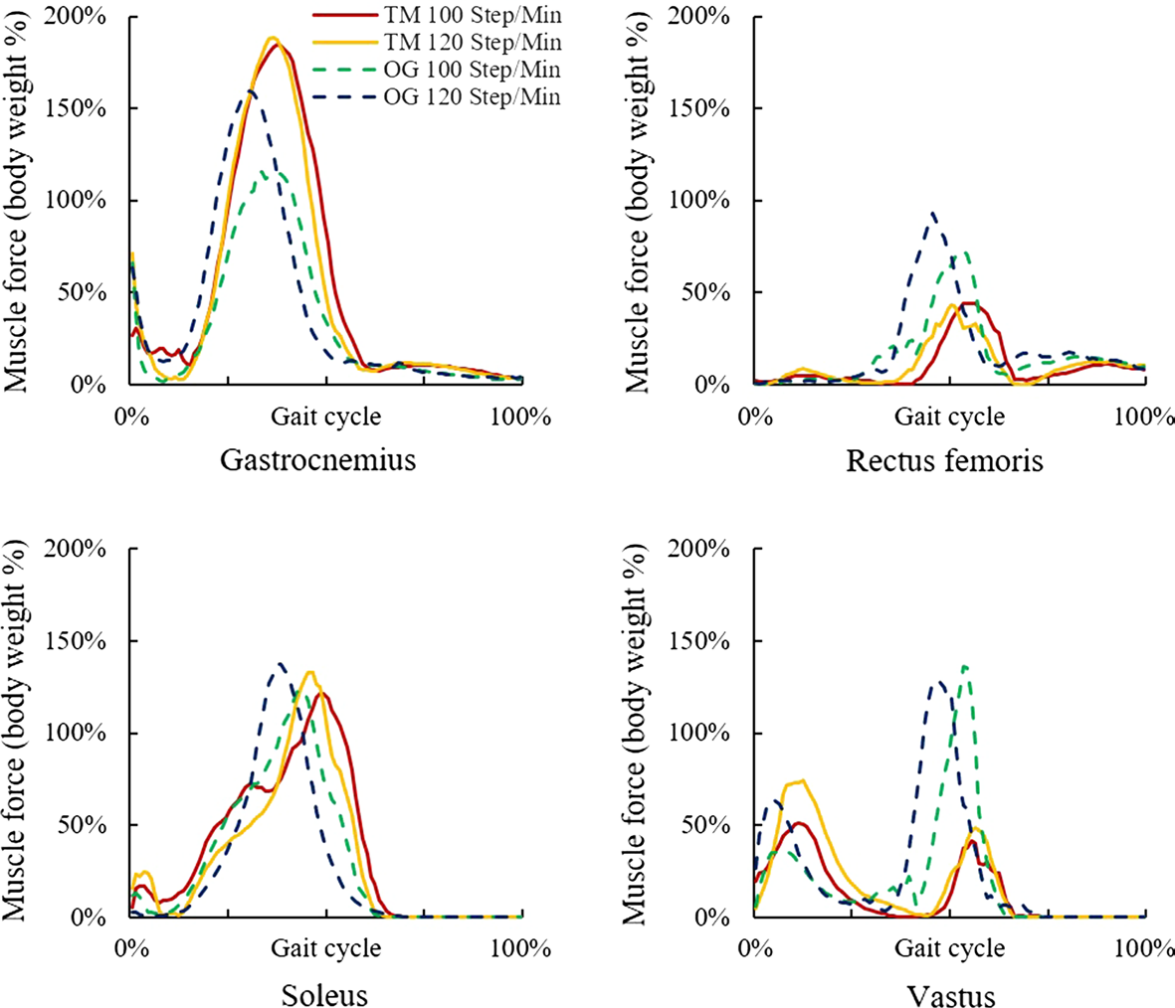
Muscle forces in treadmill and over-ground walking at 100 and 120 steps/min

In walking, the maximum Gastrocnemius force of treadmill condition was higher than that of over-ground, while the maximum forces of Rectus femoris and Vastus were lower. In treadmill walking, the maximum forces of Gastrocnemius and Rectus femoris changed slightly with the stride frequency, while the maximum forces of Soleus and Vastus increased. In over-ground walking, the maximum forces of Gastrocnemius, Rectus femoris, Soleus, and Vastus (in the initial contact phase of the gait cycle) increased with the stride frequency (Fig. 4).

**Fig. 5:**
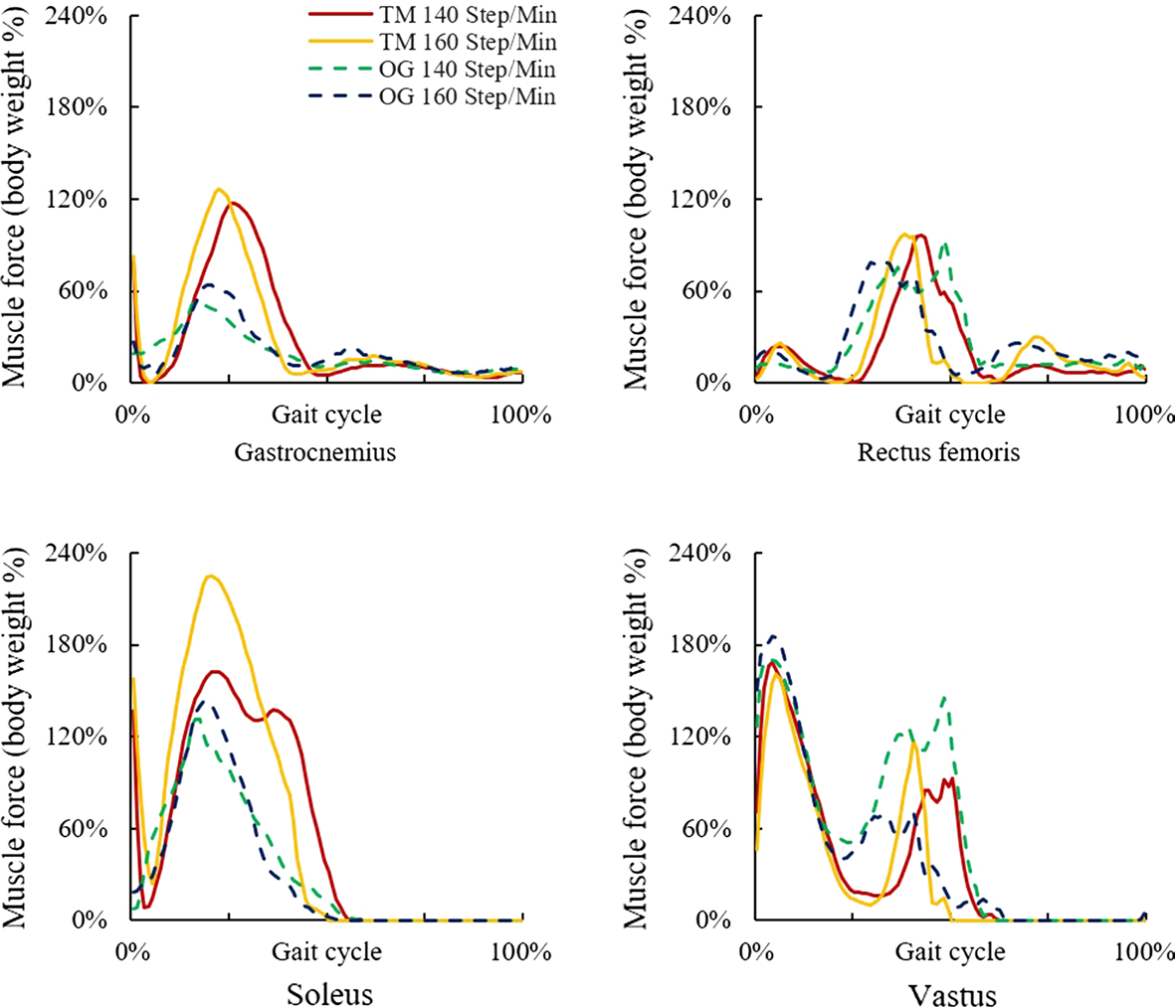
Muscle forces in treadmill and over-ground running at 140 and 160 steps/min

In running, the maximum Gastrocnemius and Soleus forces in treadmill condition were higher than those in over-ground condition. In treadmill running, the maximum forces of Gastrocnemius and Soleus increased with the stride frequency, while the maximum forces of Rectus femoris and Vastus changed slightly. In over-ground running, the maximum forces of Gastrocnemius, Soleus, and Vastus increased with the stride frequency (Fig. 5).

The averages and standard deviations of the muscle forces in treadmill and over-ground motions at each stride frequency are shown in Additional file 1. The video of inverse dynamic simulation is shown in Additional file 2.

## Discussion

The present study measured the lower limb joint motions in the treadmill and over-ground exercise with an IMU-based motion capture system. The joint motions of treadmill and over-ground conditions were similar in walking, yet different in running. Although the conclusions of previous studies are varying, these studies and our research have observed that the sagittal motion ranges of lower limb joints during treadmill running decreased to some extents compared with the over-ground running [22, 25, 26]. The effect of the stride frequency on the joint motion was also weaker. It was found that the motion range of the hip joint increased with the stride frequency in both treadmill walking and running, yet the motion range decreased in the walk-to-run transition; whereas the motion range of ankle joint remarkably increased in the walk-to-run transition, which was in agreement with the previous study [27]. Furthermore, the walk-to-run transition of ankle motion in treadmill condition was also smaller than that in over-ground condition; therefore, in terms of joint kinematics, treadmill exercise can provide smoother joint motions.

Previous studies investigated the kinetics of treadmill or over-ground exercises in terms of energy consumption, GRF, and surface EMG signals [10, 11, 21, 28]. However, these indexes cannot accurately characterize the musculoskeletal loading, which directly contributes to tissue injuries. In the present study, muscle forces were calculated with the inverse dynamic method, which has been validated in previous studies [29–31]. The calculated muscle forces were further validated with the surface EMG signals. Since the amplitude of surface EMG does not accurately reflect the muscle force [32, 33], the present study compared the timing of peak EMG signal with that of peak muscle force. A significant Spearman correlation (*R *= 0.514*, *P *= 0.03) was found, which provided the reliability of the muscle force calculation. Furthermore, the *ICC (2,k)* results of the muscle forces were greater than 0.75, which indicate desirable repeatability of the methodology.

The muscle forces between treadmill and over-ground exercise were different. Compared with the over-ground walking, Gastrocnemius (knee flexor) force in treadmill walking was greater, while the Rectus femoris and Vastus (knee extensor) forces were smaller. The result infers a smaller knee extension moment in the treadmill walking than in over-ground walking. This phenomenon was consistent with the previous study [22]. Since a smaller knee extensor contributes to smaller in situ forces in patellofemoral articular surface and anterior cruciate ligament, treadmill walking could be more suitable for the rehabilitation of people with knee degeneration and soft tissue injuries.

Compared with the over-ground running, Gastrocnemius and Soleus (plantar flexor) forces in treadmill condition were greater. The result infers a greater plantar flexion moment in treadmill running than that in over-ground running, which was in agreement with the literatures [34, 35]. An explanation is that, to adapt to the constant speed of the treadmill, neuromotor system tended to use smaller step length to facilitate the dynamic adjustment of muscle force. To maintain the running speed, Gastrocnemius force as the main driving force was remarkably increased. The finding implies that treadmill running may result in higher loading on plantar flexor, especially on gastrocnemius, which can provide basis for rehabilitation therapy customization.

In this study, the stride frequency was used as the control condition to analyze the difference between treadmill and over-ground exercises. Previous studies usually used given or self-selection speeds as the control condition. However, given the same speed, some people feel too fast while some others feel too slow. Subjects’ self-selection speed often tends to change, and its individual differences are also great. The stride frequency can be seen as the normalization of speed with the step length. Therefore, it can better serve as a control condition for gait analysis.

The present study has some limitations. First, an inverse dynamic model of human musculoskeletal system was used to investigate the differences between treadmill and over-ground exercises. However, the deformation of the bone was not considered, the muscles of upper limbs and trunk were not included, and the GRF was estimated with the GRF predict program. These factors could lead to an error in the muscle force calculation. Despite of this, based on the previous studies and the present validation, the trend of muscle forces can be evaluated and can provide a basis for the biomechanical study of the treadmill and over-ground exercise. Second, this study analyzed the joint motions in the sagittal plane; the valgus and rotation of the joints should be investigated in the future study, especially in subject of locomotor deficit. Third, the level of training will have a significant impact on the muscle response; the type of foot and shoe can influence the kinetics of walking and running. These factors should be further investigated and could be used as the adjustable parameters to manipulate the effect of exercise.

## Conclusions

This study quantified the differences of lower limb joint angles and muscle forces in treadmills and over-ground exercises. The results indicated that the joint motions of treadmill and over-ground conditions were similar in walking, yet different in running. Compared with over-ground running, the joint motion in treadmill running was smaller and less affected by stride frequency. In terms of muscle forces, treadmill running resulted in lower loading on knee extensor, yet higher loading on plantar flexor, especially on gastrocnemius. The findings and the methodology can provide the basis for rehabilitation therapy customization and sophistic treadmill design.

## Methods

### Subjects

10 healthy subjects volunteered to participate the study (5 males and 5 females, age 22.7 ± 1.2, height 1.69 ± 0.18 m, weight 63.7 ± 7.7 kg). Subjects were free from lower limb pathology as examined by physical assessment. Every subject had the experience in treadmill and over-ground running, yet not a professional runner. The study was approved by the Ethics Review Board at Beihang University. Every subject received an oral and written explanation of the study and signed an inform consent before performing the trials.

### Motion capture experiment

**Fig. 6:**
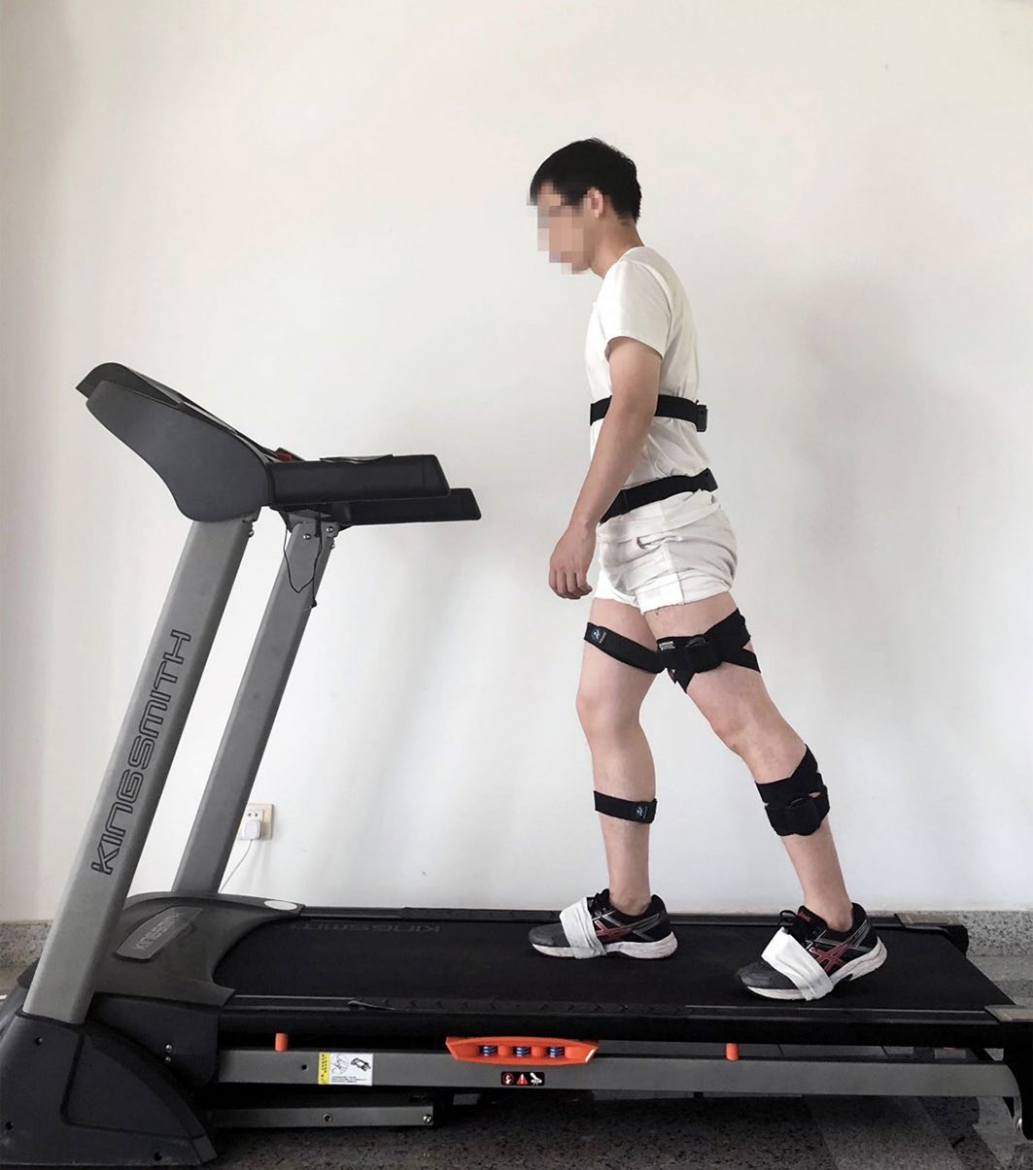
Motion capture experiment in treadmill walking. The IMU and EMG sensors were attached on the subject’s body. The data was transmitted to the computer wirelessly

The kinematical data of the subjects during treadmill and over-ground exercises were collected with the inertial-based motion capture system, MyoMotion (Noraxon, Inc., Scottsdale, USA). 8 inertial measurement unit (IMU) were attached to the lower thoracic, sacrum, thighs, shanks, and feet (Fig. 6). Each IMU consist of a three-dimensional accelerometer, gyroscope, and magnetometer, which was used to record the acceleration and rotation of the body segments and to calculate the flexion angles of hip, knee, ankle joints. Meanwhile, the surface EMG signals of lower limb muscles were measured with the wireless EMG recording system, MyoMuscle (Noraxon, Inc., Scottsdale, USA). EMG sensors were attached on the belly of four muscles: Gastrocnemius, Rectus femoris, Soleus, and Vastus. The IMU and EMG information was transmitted to the computer wirelessly. Based on the literature and pre-experiments, the stride frequency usually ranged from 103 to 122 steps/min in walking, and 132 to 182 steps/min in running [36–38]. To investigate the effect of stride frequency on walking, running, as well as walk-to-run transition, subjects were required to walk on the treadmill and ground with the frequencies of 100 and 120 steps/min, and run at 140 and 160 steps/min. A metronome App (Wuhan Net Power Technology Co., Ltd) was used to control the stride frequency. In the treadmill exercise, the kinematical data were recorded after the subject adapted to the stride frequency for 30 s. In the over-ground exercise, the subjects were required to walk or run straightly. The data were recorded after subject adapted to the stride frequency for 15 m. For each trial, 10 stable full gait cycles were extracted for further analysis. A gait cycle was defined as the period between two adjacent left foot heel-strikes. Each subject was measured for 3 times, with 5-min intervals. To eliminate the side effect of shoe type, subjects were required to wear the same type of sneakers with proper sizes.

### Inverse dynamic calculation of muscle force

**Fig. 7:**
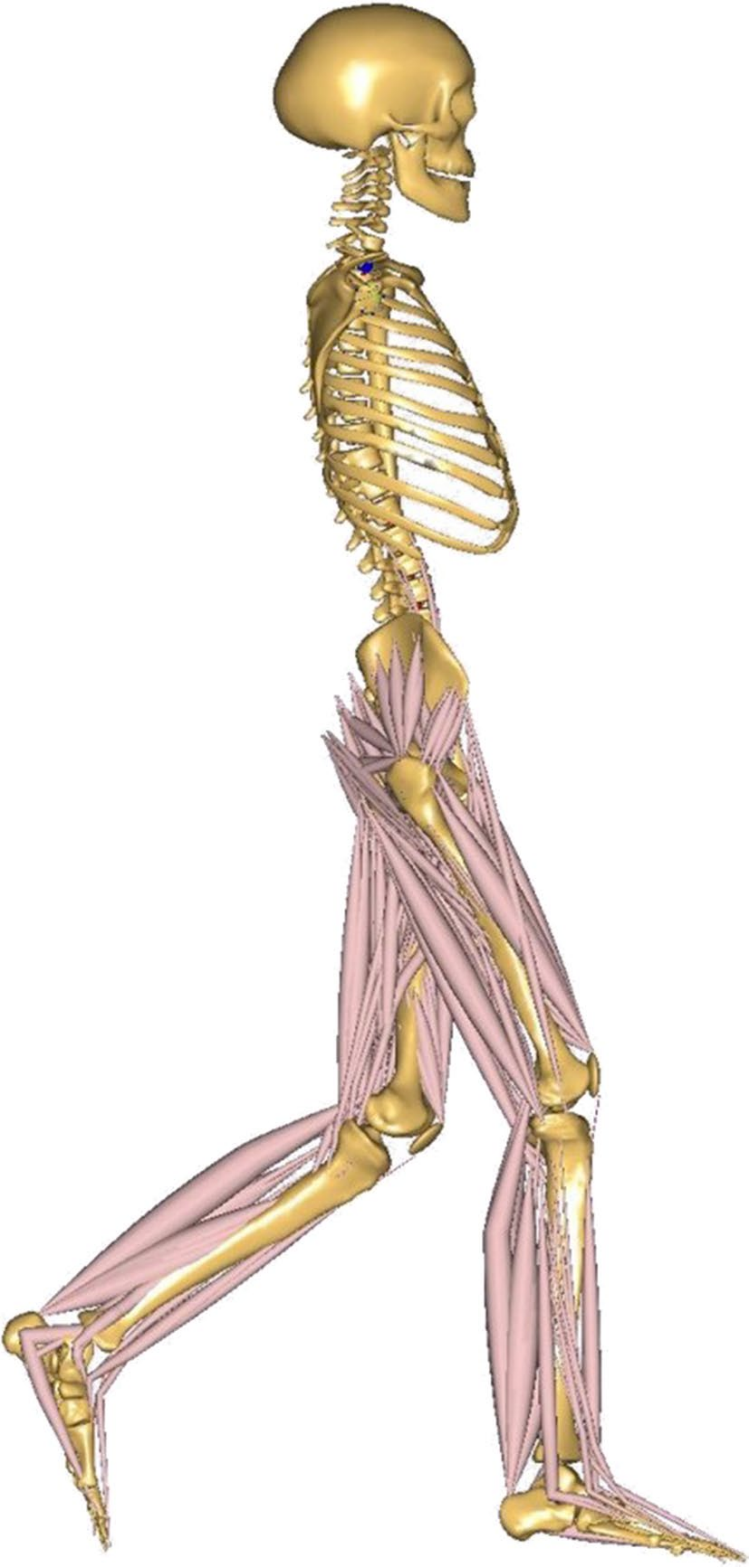
Inverse dynamic model of musculoskeletal system. Hip, knee, and ankle joints and 318 muscles were included in the lower limb part. The muscles of upper limbs and trunk were not included

The lower limb muscle forces were calculated with the inverse dynamic software, Anybody (AnyBody Technology, Aalborg, Denmark). A musculoskeletal model of human body was developed (Fig. 7). Since this study focused on the lower limb kinetics, the muscles of upper limbs and trunk were not included. Hip, knee, and ankle joints and 318 muscles were included in the lower limb model. The weight, height, lengths of thigh, shank, and foot, and the width of pelvis of each model were modified according to the corresponding subject. The joint flexion angles and body motion collected from motion capture experiment were used to drive the inverse dynamic model. A validated GRF predict program was applied to estimate the GRF during running [31].

### Data process and validation

The muscle forces were normalized with the body weight. The average of the normalized muscle forces throughout the full gait cycle was calculated. A two-way random average measure intra-class correlation coefficient (*ICC (2,k)*) was calculated to estimate the repeatability of the methodology. *ICC (2,k)* greater than 0.75 indicates a desirable repeatability. The forces of the muscles calculated with the inverse dynamic model were validated with the EMG of the muscles. The Spearman correlation is a non-parametric test for the measurement of two variables’ correlation. Compared with the Pearson correlation, Spearman correlation does not carry any assumptions about the distribution of the data. The Spearman correlation between the peak EMG signal timing and the peak muscle force timing in the gait cycle was calculated. A correlation coefficient different from 0 and a significant level (*P* value) < 0.05 indicate a considerable correlation. Statistical software SPSS (IMB, US) was used for the data analysis. Then, the effect of exercise conditions (treadmill and over-ground) and the stride frequency on the joint flexion angles and lower limb muscle forces were analyzed.

## Additional files

**Additional file 1.** The averages and standard deviations of the joint flexion and muscle forces in treadmill and over-ground motions at each stride frequency.

**Additional file 2.** Video of inverse dynamic simulation.

## Data Availability

The datasets used and/or analyzed during the current study are available from the corresponding author on reasonable request.
