# Supplementary material for: Lower limb joint motion and muscle force in treadmill and over-ground exercise
Source: Biomed Eng Online. 2019 Aug 22;18:89. doi: 10.1186/s12938-019-0708-4 (PMC6704526; doi:10.1186/s12938-019-0708-4)
Supplement: Supplementary file 1 — Additional file 1. The averages and standard deviations of the joint flexion and muscle forces in treadmill and over-ground motions at each stride frequency. [file 12938_2019_708_MOESM1_ESM.docx]

**Additional file 1. The averages and standard deviations of the joint flexion and muscle forces in treadmill and over-ground motions at each stride frequency**
