# Supplementary figures and images for: Lower limb joint motion and muscle force in treadmill and over-ground exercise
Source: Biomed Eng Online. 2019 Aug 22;18:89. doi: 10.1186/s12938-019-0708-4 (PMC6704526; doi:10.1186/s12938-019-0708-4)

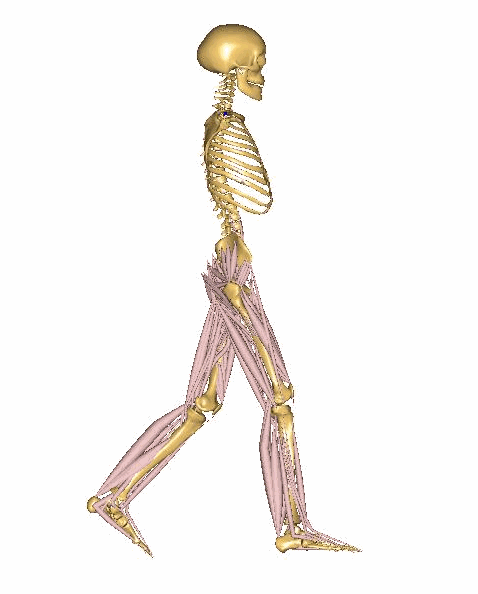

Supplement: Supplementary file 2 — Additional file 2. Video of inverse dynamic simulation. [file 12938_2019_708_MOESM2_ESM.gif]
